# CMR findings in patients with hypertrophic cardiomyopathy and atrial fibrillation

**DOI:** 10.1186/1532-429X-11-34

**Published:** 2009-09-09

**Authors:** Theano Papavassiliu, Tjeerd Germans, Stephan Flüchter, Christina Doesch, Anton Suriyakamar, Dariusch Haghi, Tim Süselbeck, Christian Wolpert, Dietmar Dinter, Stefan O Schoenberg, Albert C van Rossum, Martin Borggrefe

**Affiliations:** 1 1st Department of Medicine-Cardiology, University Hospital of Mannheim, Mannheim, Germany; 2 Department of Cardiology, VU University Medical Center, Amsterdam, the Netherlands; 3 Department of Radiology, University Hospital of Mannheim, Germany

## Abstract

**Objectives:**

We sought to evaluate the relation between atrial fibrillation (AF) and the extent of myocardial scarring together with left ventricular (LV) and atrial parameters assessed by late gadolinium-enhancement (LGE) cardiovascular magnetic resonance (CMR) in patients with hypertrophic cardiomyopathy (HCM).

**Background:**

AF is the most common arrhythmia in HCM. Myocardial scarring is also identified frequently in HCM. However, the impact of myocardial scarring assessed by LGE CMR on the presence of AF has not been evaluated yet.

**Methods:**

87 HCM patients underwent LGE CMR, echocardiography and regular ECG recordings. LV function, volumes, myocardial thickness, left atrial (LA) volume and the extent of LGE, were assessed using CMR and correlated to AF. Additionally, the presence of diastolic dysfunction and mitral regurgitation were obtained by echocardiography and also correlated to AF.

**Results:**

Episodes of AF were documented in 37 patients (42%). Indexed LV volumes and mass were comparable between HCM patients with and without AF. However, indexed LA volume was significantly higher in HCM patients with AF than in HCM patients without AF (68 ± 24 ml·m^-2 ^versus 46 ± 18 ml·m^-2^, p = 0.0002, respectively). The mean extent of LGE was higher in HCM patients with AF than those without AF (12.4 ± 14.5% versus 6.0 ± 8.6%, p = 0.02). When adjusting for age, gender and LV mass, LGE and indexed LA volume significantly correlated to AF (r = 0.34, p = 0.02 and r = 0.42, p < 0.001 respectively). By echocardiographic examination, LV diastolic dysfunction was evident in 35 (40%) patients. Mitral regurgitation greater than II was observed in 12 patients (14%). Multivariate analysis demonstrated that LA volume and presence of diastolic dysfunction were the only independent determinant of AF in HCM patients (p = 0.006, p = 0.01 respectively). Receiver operating characteristic curve analysis indicated good predictive performance of LA volume and LGE (AUC = 0.74 and 0.64 respectively) with respect to AF.

**Conclusion:**

HCM patients with AF display significantly more LGE than HCM patients without AF. However, the extent of LGE is inferior to the LA size for predicting AF prevalence. LA dilation is the strongest determinant of AF in HCM patients, and is related to the extent of LGE in the LV, irrespective of LV mass.

## Introduction

Hypertrophic cardiomyopathy (HCM) is a complex and relatively common form of genetic heart disease and the most frequent cause of sudden death in the young [1]. HCM is macroscopically characterized by -often asymmetrical - left ventricular (LV) hypertrophy in the absence of any systemic or cardiac disease likely to cause this hypertrophy. Histologically, myocyte disarray, scarring and microvascular dysfunction are the hallmarks of HCM.

Atrial fibrillation (AF) is the most common arrhythmia in HCM [2-5]. In general, AF is associated with unfavourable prognosis secondary to an increased risk of heart failure-related mortality, thrombo-embolism and severe functional impairment [6-10]. In HCM patients, increased left atrial (LA) size, advanced age and congestive heart failure symptoms have been shown to be independent predictors of the occurrence of AF [11]. However, the effect of myocardial scarring on the presence of AF has not been evaluated yet.

Cardiovascular magnetic resonance (CMR) has a high spatial resolution, is considered the gold standard for in vivo determination of left ventricular (LV) mass and volumes, and enables precise quantification of wall thickness and dimensions [12,13]. Additionally, late gadolinium-enhanced (LGE) CMR allows visualization of myocardial scarring in HCM patients [14,15].

Given the association between the extent of LGE with progressive LV dilation, ventricular arrhythmias and markers of sudden death in HCM patients we hypothesized that the extent of LGE might also be associated with the presence of AF [16-18]. Thus, we used CMR and echocardiography to evaluate whether the extent of LGE, LV and LA size, diastolic dysfunction and mitral regurgitation were associated with AF in HCM patients.

## Methods

### Study population

In total, 102 consecutive HCM patients referred for CMR were selected between February 2003 and December 2006 at 2 referral centers: 1^st ^Department of Medicine, University Hospital of Mannheim, Germany and the Department of Cardiology, VU University Medical Center, Amsterdam, the Netherlands. All HCM patients were diagnosed based on conventional criteria; left ventricular hypertrophy ≥ 15 mm on two-dimensional echocardiography in the absence of another disease that could account for the hypertrophy [19]. The work-up at intial diagnosis included electrocardiogram (ECG), echocardiography, coronary angiography, left ventriculography, 24-h Holter ECG and CMR imaging. Every 6 months the majority of patients underwent follow up visits including ECG, echocardiography and 24-h Holter ECG. Of 102 HCM patients, 15 patients were excluded due to concomitant coronary artery disease (n = 4), previous myocardial infarction (n = 3) or implantation of a pacemaker or defibrillator (n = 8), yielding a total of 87 patients included finally in this study (54 males and 33 females; mean age 58 ± 13 years). This study was approved by the institutional ethics committees of both participating medical centers and informed consent was obtained from all subjects.

### CMR acquisition

All studies were performed using a 1.5 Tesla whole body imaging system (Magnetom Sonata, Siemens Medical Systems, Erlangen, Germany). A dedicated four-element, phased-array body coil was used. Images were acquired during breath-holds in mild expiration. Scout images (coronal, sagittal and axial planes) were obtained for planning of the final double-oblique long-axis and short-axis views. To evaluate functional parameters, ECG-gated cine images were then acquired using a balanced segmented steady state free precession (trueFISP) sequence. Typical scan parameters were: 5 mm slice thickness with 5 mm interslice gap, temporal resolution 35 ms, repetition time 3.2 ms, echo time 1.2 ms, flip angle 60 degrees, and typical in-plane spatial resolution 1.4 × 1.8 mm^2^. After obtaining standard 4, 3 and 2 chamber long axis cines, a stack of 9 to 12 short-axis slices was used for full coverage of the left and right ventricle. The LGE images were obtained 10-15 min after intravenous administration of 0.2 mmol·kg^-1 ^gadolinium-DTPA (Magnevist, Schering AG, Berlin, Germany), using an inversion recovery turbo Fast Low Angle Shot (FLASH) sequence with 6 mm slice thickness at the same position as the long- and short-axis cines in end diastole [20]. The inversion time was adjusted per patient to optimally null signal from normal myocardium typically between 250 and 300 ms. Total acquisition time averaged 40 min.

### Left ventricular and left atrial image analysis using CMR

LV end diastolic volumes, LV end-systolic volumes, LV stroke volume, LV ejection fraction and LV myocardial mass were assessed off-line from the serial short-axis true FISP cine loops using dedicated commercially available software (ARGUS, Siemens, Erlangen, Germany). In addition to volumetric measurements, one-dimensional measurements of LV end diastolic dimensions (LVEDD), posterior wall thickness (PWT) and maximum interventricular septum wall thickness (SWT) were measured from end diastolic short-axis views. LA volumes were measured at end systole by the biplane area-length method on 4 and 2 chamber long axis views [21].

The extent of LGE was quantified using semi-automatic commercially available software (MASS, Medis, Leiden, the Netherlands) defining a signal intensity over 2 standard deviations above the mean signal intensity of a region of interest drawn in remote myocardium in the same slice as where the LGE was present [14]. The extent of LGE was then expressed as a percentage of the total LV mass to enable comparison of LGE burden between patients with different LV mass. LV and LA dimension and extent of LGE were measured by 2 observers blinded to all clinical patient details (TP; TG).

### Echocardiography

Standard echocardiographic images were obtained using a Vivid 7 machine (GE Ultrasound, Horton, Norway) with a 2.5 MHz phased array transducer at end-expiratory apnea.

Peak velocity of early (E) and late (A) wave of transmitral flow and E-wave deceleration time (DT) were measured from the pulsed-wave Doppler (PWD) obtained at the tip of mitral leaflets. Tissue Doppler imaging (TDI) was used to obtain early diastolic LV myocardial velocities (E') in the apical 4- and 2-chamber views with a 2 mm sample volume placed at the lateral, septal, anterior, and inferior mitral annulus. The average of the four annular sites (E'_global_) was used. All echocardiographic measurements were averaged over three consecutive cardiac cycles, measured by a single investigator blinded to all other variables. LV diastolic dysfunction was defined as follows: [1] PWD criteria: E/A ratio <1 if age <55 or <0.8 if age ≥ 55, and/or DT >240 ms; [2] TDI criteria: E'_global _≤ 12.9 cm/s if age <40; E'_global _≤ 10.2 cm/s if age 40-59; and E'_global _≤ 7.2 cm/s if age ≥ 60. Color Doppler flow imaging was used for semiquantitative assessment of mitral regurgitation.

### Atrial Fibrillation

AF was documented based on ECG recordings obtained when patients presented with acute onset of symptoms or during follow up visits including 24 hour ambulatory Holter ECG monitoring. AF was classified into paroxysmal and persistent. AF was considered paroxysmal when self-terminating and persistent when sustained over 7 days. In our study only patients were included with episodes of atrial fibrillation of at least 1 hour.

### Statistical Analysis

All data are presented as a mean ± standard deviation. For comparing LV ejection fraction, LV end-systolic volume, LA volume, LV mass, and percentage of LGE between HCM patients with and without AF, an unpaired, 2-tailed student's t-test was used. A Chi-square test was used to evaluate if the presence of LGE and symptoms were different between HCM patients with and without AF. To assess differences in LV and LA dimensions and extent of LGE between asymptomatic (NYHA I) and symptomatic HCM patients (NYHA II-IV), an unpaired, 2-tailed student's t-test was also used. Pearson's correlation was used to correlate extent of LGE with LA volume and LV mass. Partial correlation was used to correct the correlation between the extent of LGE and LA volume with presence of AF for age, gender and LV mass. Multivariate analysis was performed with logistic regression analysis using block entry of the following variables: LA volume, extent of LGE, LV mass, presence of mitral regurgitation, presence of diastolic dysfunction, age and gender to evaluate if these variables were independent predictors of AF, provided to have a p < 0.10 in univariate analysis. Area-under-curve analysis was performed to evaluate if LA volume, extent of LGE and LV mass would increase the likelihood of having AF in HCM patients. Additionally, receiver-operator-curve analysis was used to determine the optional cut-off value of LA volume and extent of LGE to discriminate patients at increased risk of AF from patients without. All results were considered statistically significant when p < 0.05. Analyses were performed with Statistical Package for Social Sciences (SPSS for windows 14.0, Chicago, IL, USA).

## Results

In 87 of included HCM patients, AF was documented in 37 HCM patients (43%) at time of initial diagnosis or during follow-up. Baseline characteristics are presented in table 1. AF was classified as paroxysmal in 27 patients (31%) and persistent in 10 patients (12%). According to the NYHA classification system, 41 (47%) were asymptomatic (NYHA functional class I) and the other 46 (53%) were symptomatic (NYHA functional classes II-IV). By echocardiographic examination, LV diastolic dysfunction was evident in 35 (40%) patients. Mitral regurgitation greater than II was observed in 12 patients (14%). A total of 45 patients (52%) were taking beta-blockers, 16 (18%) calcium-channel blockers, 9 (10%) amiodarone, 3 (3%) digitalis and 1 (1%) flecainide. A similar proportion of patients in the AF and non AF subgroups were taking cardioactive medications.

**Table 1:** Patient Demographics and Baseline Characteristics.

	**All Patients (n = 87)**	**Patients with AF (*n *= 37)**	**Patients without AF (n = 50)**
Age (years)	58 ± 13	59 ± 15	56 ± 11
Male gender	54 (61%)	22 (59%)	32 (64%)
Hypertension	8 (9%)	3(8%)	5(10%)
Diabetes mellitus	6 (7%)	3(8%)	3(6%)
*NYHA functional class*			
I	41 (47%)	17(46%)	24(48%)
II	38 (44%)	16(43%)	22(44%)
III/IV	8 (9%)	4(10%)	4(8%)
Family history of HCM or sudden death	20 (23%)	9(24%)	11(22%)
Syncope	6 (7%)	4(10%)	2(4%)
*Atrial fibrillation*	37 (43%)		
None	50(57%)	0	50(100%)
Paroxysmal	27(31%)	27 (73%)	0
Persistent	10(12%)	10 (27%)	0
*Echocardiographic data*			
LV diastolic dysfunction, n (%)	35 (40%)	22 (59%)	13 (26%)
Mitral regurgitation			
1	27 (31%)	15 (40%)	12 (24%)
≥ 2	12(14%)	7 (18%)	5 (10%)
*Medications, n (%)*			
Beta-blocker	45(52%)	20 (55%)	25 (49%)
Calcium-channel blocker	16(18%)	7 (18%)	9 (18%)
Amiodarone	9(10%)	5(13%)	4 (8%)

Data are presented as the mean value ± SD or number (%) of subjects. NYHA = New York Heart Association; HCM = hypertrophic cardiomyopathy; LV = left ventricular

Age, LV ejection fraction, LV end systolic volume, and LV mass were all comparable between HCM patients with and without AF. However, LV end systolic volume tended to be larger and LV ejection fraction tended to be lower in HCM patients with AF, see table 2. LA volume was significantly larger in HCM patients with AF than in patients without AF (p = 0.0002). In symptomatic HCM patients, LV end systolic volume (p = 0.04), LA volume (p = 0.03) and extent of LGE (p = 0.03) were significantly larger compared to asymptomatic HCM patients, see table 3. AF occurred in 41% of asymptomatic HCM patients and in 43% of symptomatic HCM patients, which was not significant. In symptomatic patients the percentage of persistent AF tended to be higher compared to asymptomatic patients (17% versus 5%, p = 0.13).

**Table 2:** LV and LA dimensions and extent of late gadolinium enhancement in HCM patients.

	**Atrial fibrillation (*n *= 37)**	**No atrial fibrillation (*n *= 50)**	**p-value**
Age (years)	59 ± 15	56 ± 11	0.40
LV ejection fraction (%)	56 ± 10	61 ± 10	0.07
LV mass (gr·m^-2^)	97 ± 31	99 ± 34	0.76
LV EDD (mm)	51 ± 7	49 ± 8	0.51
SWT (mm)	19 ± 6	18 ± 4	0.28
PWT (mm)	10 ± 3	11 ± 3	0.28
LVEDV (mL·m^-2^)	86 ± 23	79 ± 20	0.16
LVESV (mL·m^-2^)	37 ± 14	31 ± 11	0.07
LVSV (mL·m^-2^)	49 ± 15	50 ± 15	0.89
LA volume (mL·m^-2^)	68 ± 24	46 ± 18	0.0002
LGE (%)	12.4 ± 14.5	6.0 ± 8.6	0.02

Data are presented as ± standard deviation. Volumes are indexed to body surface area. EDD = end diastolic dimension, EDV = end diastolic volume, ESV = end systolic volume, LA = left atrial, LGE = late gadolinium enhancement, LV= left ventricular, PWT = posterior wall thickness, SWT = septal wall thickness

**Table 3:** Comparison of left ventricular and left atrial dimensions between asymptomatic and symptomatic HCM patients.

	**Asymptomatic HCM (n = 41)**	**Symptomatic HCM (n = 46)**	**p-value**
Atrial fibrillation	17 (41%)	20(43%)	0.68
None	24(59%)	26(57%)	0.97
Paroxysmal	15(37%)	12(26%)	0.41
Persistent	2(5%)	8(17%)	0.13
LV ejection fraction (%)	60 ± 10	58 ± 9.5	0.32
LV mass (gr·m^-2^)	99 ± 30	97 ± 34	0.84
LV end systolic volume (ml·m^-2^)	29 ± 13	37 ± 13	0.04
LA volume (ml·m^-2^)	48 ± 21	59 ± 23	0.03
Extent of LGE (%)	6.0 ± 9.0	11 ± 13	0.03

Data are presented as ± standard deviation. Volumes are indexed to body surface area. HCM = hypertrophic cardiomyopathy patients, LA = left atrial. LGE = late gadolinium enhancement, LV = left ventricular

In general, in our collective LGE occurred in a patchy distribution with multiple foci within hypertrophied regions of the intervenricular septum, the anterior and posterior walls. In 14 patients LGE was limited at the RV insertion points (16/59 = 27%). In two patients LGE was also present in the lateral wall (2/59 = 3%). In 13 patients LGE (13/59 = 22%) was diffuse trans-septal and RV septal. In these patients, 10 had AF. However, the small sample size precludes any firm conclusions.

The mean extent of LGE was higher in HCM patients with AF compared to those without (12.4 ± 14.5% versus 6.0 ± 8.6%, p = 0.02). However, the prevalence of AF was comparable between HCM patients with LGE to those without LGE (27/59, 46% versus 10/28, 35%, p = 0.5). When adjusting for age, gender and LV mass, both the extent of LGE and LA volume moderately correlated to AF (r = 0.34, p = 0.02 and 0.42, p < 0.001 respectively). Interestingly, LA volume moderately correlated to the extent of LGE (r = 0.31, p = 0.035), irrespective of LV mass, which did not correlate with LA volume (r = 0.08, p = 0.69). LV mass also did not correlate with the extent of LGE (r = 0.024, p = 0.83).

LA volume was significantly larger in HCM patients with dystolic dysfunction than in patients without diastolic dysfunction (p = 0.04). In HCM patients with diastolic dysfunction the mean extent of LGE tended to be higher compared to HCM patients without diastolic dysfunction. However, this comparison did not reach a significant level (13% versus 9%, p = 0.3).

With multivariate logistic regression analysis, a model using the extent of LGE, LA volume, presence of mitral regurgitation and presence of diastolic dysfunction to predict AF in HCM patients had an r-square of 0.31, p < 0.01, and revealed that only LA volume and diastolic dysfunction were independently associated with a history of AF in HCM patients (p = 0.006, p = 0.01 respectively).

**Figure 1:**
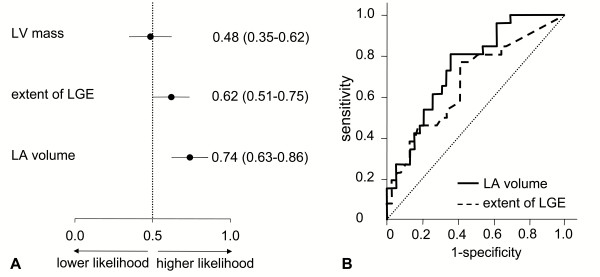
**Receiver operating characteristic (ROC) curve analysis of left ventricular mass, extent of late gadolinium enhancement (LGE) area and left atrial volume as a predictor of AF**. In the panel A, mean area-under-curve with 95% confidence intervals are presented. In the panel B, ROC curves are presented of extent of LGE and left atrial volume to determine optimal cut-off values. LA = left atrial, LV = left ventricular, LGE = late gadolinium enhancement.

The univariate association between the presence of AF with LA volume and extent of LGE was confirmed with ROC analysis, see figure 1. The receiver-operator curve indicates that cut-off value for LA volume of 49.8 ml/m^2 ^has optimal discriminative power to predict AF, yielding a sensitivity of 80.8% and specificity of 64.1%. With respect to the extent of LGE, an optimal cut-off value of 3.3% was found to have a sensitivity of 76.9% and a specificity of 59.0%, see figure 1.

## Discussion

The present CMR study demonstrates that HCM patients with AF had larger LA volumes and displayed more LGE than HCM patients without AF. LA volume and diastolic dysfunction were independently associated with a history of AF in HCM patients. However, among these two parameters LA volume was the strongest independent determinant of AF. Additionally, LA volume moderately correlated to the extent of LGE irrespective of LV mass. Also, in symptomatic HCM patients in this study, LV end-systolic volumes and LA volumes were larger and extent of LGE was higher compared to asymptomatic HCM patients, which is in line with previous reports [16,22]. Interestingly, paroxysmal AF did not seem to be more prevalent in symptomatic HCM patients than asymptomatic HCM patients, but persistent AF tended to have a higher prevalence in symptomatic HCM patients compared to asymptomatic HCM patients.

AF is a commonly reported complication of HCM [2,4,8,10]. Established AF is uncommon in the young, but in adults its prevalence can reach up to 30% [23]. Remarkably, the prevalence of AF in our study population was higher than previously reported. This may in part be due to the large number of follow-up visits, ECGs and 24 hours ambulatory Holter ECG recordings in the majority of patients. Since AF is considered a key determinant of HCM-related morbidity and mortality [7,10-12], the identification of predictors of AF is of paramount clinical importance.

In a community-based HCM population, Olivotto et al. found that the strongest predictor of AF was increased LA volume, independent of age and functional NYHA functional class [11]. In addition to LA volume, Losi et al. reported that LA fractional shortening and age were independent predictors for the development of AF in HCM patients [23]. However, interpretation of these studies may be difficult, while LA volume measurement may be hindered by a poor acoustic window which is inherent to echocardiography. By using CMR, we merely omitted these limitations to measure LA volume, and confirmed the importance of LA volume in relation to AF in HCM patients. In this CMR study, a LA volume cut-off value of 50 ml·m^-2 ^had an optimal discriminative power to predict AF, yielding a sensitivity of 81% and a specificity of 64%. This cut-off value is comparable with the results from Losi et al, who found a cut-off value of 44 ml·m^-2 ^determined with echocardiography [23]. The strong relation between LA dilation and AF in HCM patients may be explained by the electrical and structural remodeling that occur in the process of LA dilation, including shortening of the atrial effective refractory period and local conduction delay [24].

In addition, AF was associated with LGE in HCM patients. However, not the presence but the extent was shown to be indicative for AF. This is in line with the findings of a necropsy study of HCM patients performed by Yamaji et al [25], who found that the extent of LV fibrosis was significantly higher in HCM patients with AF (24.4 ± 0.6%) than without AF (17.6 ± 0.7%). The total fibrotic burden in the LV found by Yamaji et al was higher compared to our findings. This difference might be explained by the fact that patients in this necropsy study died from progressive HCM, which has been associated with an increased fibrotic burden in comparison with milder forms of HCM [16]. Moreover, systematic underestimation of fibrosis quantification is an important limitation of LGE imaging, by which only focal, but not diffuse areas of fibrosis can be visualized and quantified. In addition, the LV must remain in diastasis for at least 100 ms for optimal quality of the LGE images. Therefore, in this study, only HCM patients with AF who had acceptable rate control were referred for CMR, which may have introduced a selection bias into our analysis.

The underlying mechanisms relating LV LGE with AF are unknown. The histological background of LGE in HCM patients has been suggested to be myocardial scarring and/or interstitial fibrosis [15]. Focal areas of myocardial scarring have been related to ventricular arrhythmias, but may also serve as anatomical substrates for AF [17,18]. Interstitial fibrosis is produced by fibroblasts that are activated by cardiotrophic mediators, such as angiotensin II. Interstitial fibrosis together with impaired relaxation of the cardiomyocyte through altered Ca^2+^-handling may ultimately lead to diastolic dysfunction of the LV and subsequent LA dilation and explain the relation between the extent of LGE, LA dilation and subsequent AF [26,27]. This statement was supported by the moderate, but significant correlation between LGE and LA volume found in this study. Additionally, patients with diastolic dysfunction revealed considerably more LGE than patients without, indicating a potential relationship between LGE and diastolic function.

**Figure 2:**
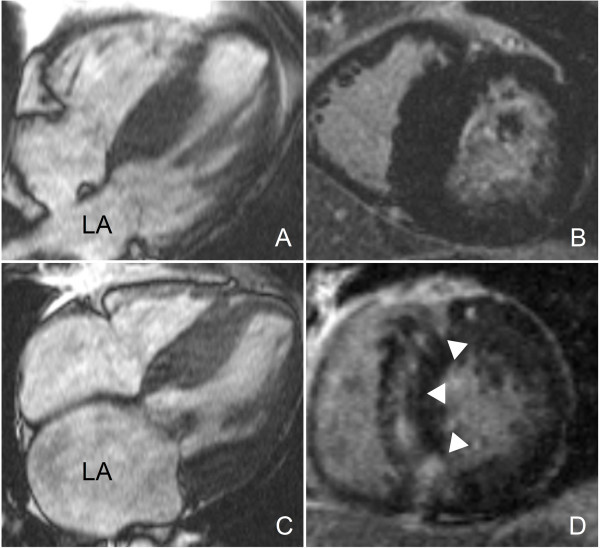
**HCM patients with comparable left ventricular mass and different left atrial size**. **A, C. End diastolic 4 chamber cine images**. Note that the left atrium of the first patient (A) is smaller compared to the left atrium of the second patient (C), despite comparable LV mass. The end diastolic short axis LGE images (**B, D**) reveal that the first patient (B) has no LGE, while the second patient (D) has extensive LGE of the septum, as indicated by the white arrowheads.

In contrast to patients with hypertension and in normal population [28,29], LV mass did not correlate to LA dilation in these HCM patients, see figure 2. In HCM patients, mitral regurgitation, originating from systolic anterior motion of the mitral valve leaflet due to LV outflow tract obstruction and increased interstitial fibrosis and myocardial scarring also importantly attribute to increased LA volume. However, no quantitative data evaluating the relative contribution of the extent of LGE, LV diastolic dysfunction and mitral regurgitation to LA dilation in HCM patients are yet available.

Mitral regurgitation is very common in patients with HCM. Also, in our study population mitral regurgitation was present in 39 (45%) patients. However, mitral regurgitation was not a independent predictor of AF in HCM patients. Similar to our results, Losi et al. found that mitral regurgitaiton was not a determinant of AF in any model of multivariate analysis [23].

### Clinical implication

Should further studies confirm the pathogenic role of fibrosis in diastolic dysfunction and subsequent LA dilation in HCM, different treatment options aimed at stopping or even reversing the process of fibrosis could gain clinical importance. For example, angiotensin II blockade, statins and aldosteron antagonists, have already been demonstrated experimentally to cause regression of fibrosis, hypertrophy and disarray in animal HCM models [30-32]. Moreover, the strong relation between LA dilation and AF may justify to use LA size > 50 mL·m^-2 ^determined with CMR as an indication to aggressively search for the presence of AF.

### Limitations

For cine and LGE imaging, we used a gap of 5 and 4 mm between slices for full coverage of the left ventricle, thereby omitting 40 to 50% of total myocardium. This may potentially have introduced inaccuracy of volume and mass measurement; however, in a comparative study performed by Hogan et al, contiguous acquisition of slices and acquisition with 4 mm showed comparable accuracy and yielded comparable volumes [33]. As mentioned previously, LGE imaging systemically underestimates total burden of diffuse fibrosis, but a recent CMR study has shown promising results to overcome this limitation [34]. In addition, the temporal resolution of CMR cine imaging was not sufficient to adequately assessed LV diastolic dysfunction. Therefore we used the echocardiographic data to evaluate the diastolic function.

In conclusion, HCM patients with a history of AF display significantly more LGE than HCM patients without AF. However, the extent of LGE is inferior to the LA size for predicting AF prevalence. LA dilation is the strongest determinant of AF in HCM patients, and is related to the extent of LGE in the LV, irrespective of LV mass.

## Abbreviations

CMR: cardiovascular magnetic resonance; AF: atrial fibrillation; LGE: late gadolinium enhancement; HCM: hypertrophic cardiomyopathy; EF: ejection fraction; LV: left ventricular; LA: left atrium; trueFISP: Balanced segmented steady state free precession.

## Competing interests

The authors declare that they have no competing interests.

## Authors' contributions

TP performed and evaluated CMR images, participated in study-design, figures and tables and drafted the manuscript. TG contributed equally to the writing of this manuscript. SF participated in study-design and evaluation of CMR data. CD participated in the revision of the manuscript and the evaluation of the echocardiograpical data. AS participated in study design and its coordination. DH participated in collection and evaluation of clinical data. TS participated in study-design and revision of the manuscript. CW participated in study-design and statistical analysis. DD participated in study-design and evaluation of CMR-images. SS participated in study-design and evaluation of CMR-images. AvR was responsible for the revision of the manuscript. MB Participated in study-design, scientific and clinical advice concerning HCM and was responsible for the revision of the manuscript. All authors read and approved the final manuscript.
